# Identification of *Nicotiana tabacum* Linkage Group Corresponding to the Q Chromosome Gene(s) Involved in Hybrid Lethality

**DOI:** 10.1371/journal.pone.0037822

**Published:** 2012-05-22

**Authors:** Takahiro Tezuka, Chihiro Matsuo, Takahiro Iizuka, Masayuki Oda, Wataru Marubashi

**Affiliations:** 1 Graduate School of Life and Environmental Sciences, Osaka Prefecture University, Sakai, Osaka, Japan; 2 School of Life and Environmental Sciences, Osaka Prefecture University, Sakai, Osaka, Japan; 3 School of Agriculture, Meiji University, Kawasaki, Kanagawa, Japan; Nanjing Forestry University, China

## Abstract

**Background:**

A linkage map consisting of 24 linkage groups has been constructed using simple sequence repeat (SSR) markers in *Nicotiana tabacum*. However, chromosomal assignments of all linkage groups have not yet been made. The Q chromosome in *N. tabacum* encodes a gene or genes triggering hybrid lethality, a phenomenon that causes death of hybrids derived from some crosses.

**Methodology/Principal Findings:**

We identified a linkage group corresponding to the Q chromosome using an interspecific cross between an *N. tabacum* monosomic line lacking the Q chromosome and *N. africana*. *N. ingulba* yielded inviable hybrids after crossing with *N. tabacum*. SSR markers on the identified linkage group were used to analyze hybrid lethality in this cross. The results implied that one or more genes on the Q chromosome are responsible for hybrid lethality in this cross. Furthermore, the gene(s) responsible for hybrid lethality in the cross *N. tabacum* × *N. africana* appear to be on the region of the Q chromosome to which SSR markers PT30342 and PT30365 map.

**Conclusions/Significance:**

Linkage group 11 corresponded to the Q chromosome. We propose a new method to correlate linkage groups with chromosomes in *N. tabacum*.

## Introduction

Hybrid lethality is a phenomenon that causes death of hybrids, and is one of the postzygotic mechanisms of reproductive isolation. This phenomenon, also called hybrid weakness or hybrid necrosis, is observed in plant species such as *Nicotiana* species [1], rice [2], wheat [3], *Gossypium* species [4] and *Arabidopsis thaliana* [5]. Hybrid lethality is considered to play a crucial role in the evolution of plants. An example supporting this hypothesis is that hybrid seedlings between two sympatric species, *Papaver dubium* and *P. rhoeas*, die, indicating that hybrid lethality functions as a barrier to gene flow between these species [6]. However, hybrid lethality can be an obstacle when desirable genes are to be introduced into cultivated species in breeding programs.

The genus *Nicotiana* includes 76 species classified into 13 sections [7]. Only *N. tabacum* (2n = 48, SSTT) and *N. rustica* are cultivated species, while the others are wild species. These cultivated species, especially *N. tabacum*, which belongs to section *Nicotiana*, have been crossed with many wild species. In several interspecific crosses, however, hybrid seedlings show hybrid lethality and die within a few weeks or months [1], [8]. The hybrid lethality phenotype is specific to particular cross combinations in *Nicotiana*. Hybrid lethality in this genus is classified into five types based on the observed phenotype: Type I, browning of shoot apex and root tips; Type II, browning of hypocotyl and roots; Type III, yellowing of true leaves; Type IV, formation of multiple shoots; and Type V, fading of shoot color [1], [9].

In previous studies, we focused on crosses between *N. tabacum* and species in *Nicotiana* section *Suaveolentes*. The section *Suaveolentes* includes 25 species restricted to Australasia and one African species, *N. africana*, which is the only known species in Africa [7]. These species are geographically isolated from the majority of species in other sections, most of which are distributed in the Americas. All species in section *Suaveolentes* are allotetraploids, and the section is considered to have originated from a single polyploidization event approximately 10 Mya, followed by speciation to produce the species known today [10]. Based on sequence analyses of nuclear-encoded, chloroplast-expressed glutamine synthetase (ncpGS), it has been suggested that the maternal progenitor is *N. sylvestris* (2n = 24, SS; section *Sylvestres*) and the paternal progenitor is *Nicotiana* section *Trigonophyllae* (2n = 24) [11]. *N. tabacum* is another allotetraploid that originated by interspecific hybridization of *N. sylvestris* with *N. tomentosiformis* (2n = 24, TT; section *Tomentosae*) with subsequent chromosome doubling approximately 200,000 years ago [10]–[13].

We crossed 11 species of section *Suaveolentes* with *N. tabacum*, and demonstrated that inviable hybrids were produced after crosses using nine species: *N. africana*, *N. debneyi*, *N. excelsior*, *N. goodspeedii*, *N. gossei*, *N. maritima*, *N. megalosiphon*, *N. suaveolens* and *N. velutina* [14]–[16]. The hybrid lethality observed in all these crosses at 28°C was identified as Type II, characterized by browning of hypocotyl and roots and suppression of symptoms at elevated temperatures ranging from 34 to 36°C. Our results also indicated that one or more genes on the Q chromosome in the S subgenome of *N. tabacum* are responsible for hybrid lethality in these crosses. Exceptionally, only *N. benthamiana* and *N. fragrans*, which belong to section *Suaveolentes*, produced 100% viable hybrids after crossing with *N. tabacum* [16], [17].

Molecular markers would be useful to identify the location of the gene(s) responsible for hybrid lethality on the Q chromosome. Previously, we developed random amplified polymorphic DNA (RAPD) markers, inter-simple sequence repeat (ISSR) markers and sequence tagged site (STS) markers, all of which are specific to the Q chromosome [14], [18]. However, the locations of these markers on the Q chromosome are unclear. Recently, the first linkage map of *N. tabacum* was constructed using simple sequence repeat (SSR) markers [19]. This map consists of 24 linkage groups, although three putative linkage groups (3a/3b, 8a/8b and 14a/14b) are each divided into two sublinkage groups. More recently, a high density linkage map containing 24 linkage groups has been developed using 2317 SSR markers [20]. Chromosomal assignments of the linkage groups have not yet been made, except for the A chromosome, as described in the Discussion.

Each chromosome of *N. tabacum* is lettered alphabetically (A to Z, excluding X and Y); chromosomes A to L belong to the T subgenome and M to Z to the S subgenome. A complete set of 24 monosomic lines of *N. tabacum* (Haplo-A to Z) has been established in the genetic background of ‘Red Russian’ [21], [22]. When the above-mentioned nine species of section *Suaveolentes* are crossed with Haplo-Q, two types of hybrid seedlings are obtained: inviable hybrid seedlings possessing the Q chromosome and viable hybrid seedlings lacking the Q chromosome [14]–[16]. Using hybrids grown at 36°C, a linkage group corresponding to the Q chromosome can be identified from the linkage map of Bindler et al. [20].

In the present study, we identified a linkage group corresponding to the Q chromosome using hybrid seedlings from the cross Haplo-Q × *N. africana* by the Michelmore et al. method of bulked segregant analysis [23]. SSR markers on the identified linkage group were used to investigate Q chromosome involvement in hybrid lethality observed in the cross *N. tabacum* × *N. ingulba*. Furthermore, we estimated the location of the gene(s) triggering hybrid lethality using a Haplo-Q × *N. africana* hybrid with a possible deletion of the Q chromosome segment.

## Results

### Preparation of DNA Bulks

Two types of DNA bulks, which differed in the presence or absence of the Q chromosome, were prepared using crosses *N. tabacum* ‘Red Russian’ × *N. africana* and *N. tabacum* Haplo-Q × *N. africana*. Five and 21 hybrid seedlings were obtained from F_1_ seeds of the two respective crosses. All hybrid seedlings were cultured at 36°C, a temperature at which hybrid lethality is suppressed [16]. Four Q-chromosome-specific STS markers, QCS1, QCS2, QCS3 and QCS4, were used to determine whether the Q chromosome was present in these hybrid seedlings. All markers were detected in all five hybrid seedlings from the cross ‘Red Russian’ × *N. africana* and five of the hybrid seedlings from the cross Haplo-Q × *N. africana*, but not in the other 15 hybrid seedlings from that cross (Table 1). The presence or absence of the Q chromosome was also confirmed by viability of the hybrid seedlings. When the hybrid seedlings cultured at 36°C were transferred to 28°C, the seedlings in which the markers were detected died, while those in which the markers were not detected survived. Two DNA bulks (Q+1 and Q+2) were prepared from hybrid seedlings possessing the Q chromosome and two DNA bulks (Q–1 and Q–2) were prepared from hybrid seedlings lacking the Q chromosome (Table 1).

**Table 1:** Two types of DNA bulks prepared from DNA of hybrids from crosses *N. tabacum* × *N. africana*.

			Number of hybrids		
DNA bulk	Cross combination	Q-chromosome-specific STS markers^a^	Total	Inviable	Viable
Q+1	‘Red Russian’ × *N. africana*	+	5	5	0
Q+2	Haplo-Q × *N. africana*	+	5	5	0
Q–1	Haplo-Q × *N. africana*	–	8	0	8
Q–2	Haplo-Q × *N. africana*	–	7	0	7
–	Haplo-Q × *N. africana*	±	1	0	1

a A ‘+’ indicates that all STS markers, QCS1, QCS2, QCS3 and QCS4, were detected and a ‘–’ indicates that they were not. A ‘±’ indicates that QCS1, QCS3 and QCS4 were detected but QCS2 was not detected.

Among 21 hybrid seedlings from the cross Haplo-Q × *N. africana*, we found one hybrid seedling in which QCS1, QCS3 and QCS4 were detected and QCS2 was not detected (Table 1). This hybrid seedling was viable after transfer to 28°C, suggesting that deletion of the Q chromosome region containing the gene(s) triggering hybrid lethality occurred. This hybrid seedling was used to determine the chromosomal region of the gene(s) triggering hybrid lethality as described below.

### Identification of a Linkage Group Corresponding to the Q Chromosome

**Figure 1:**
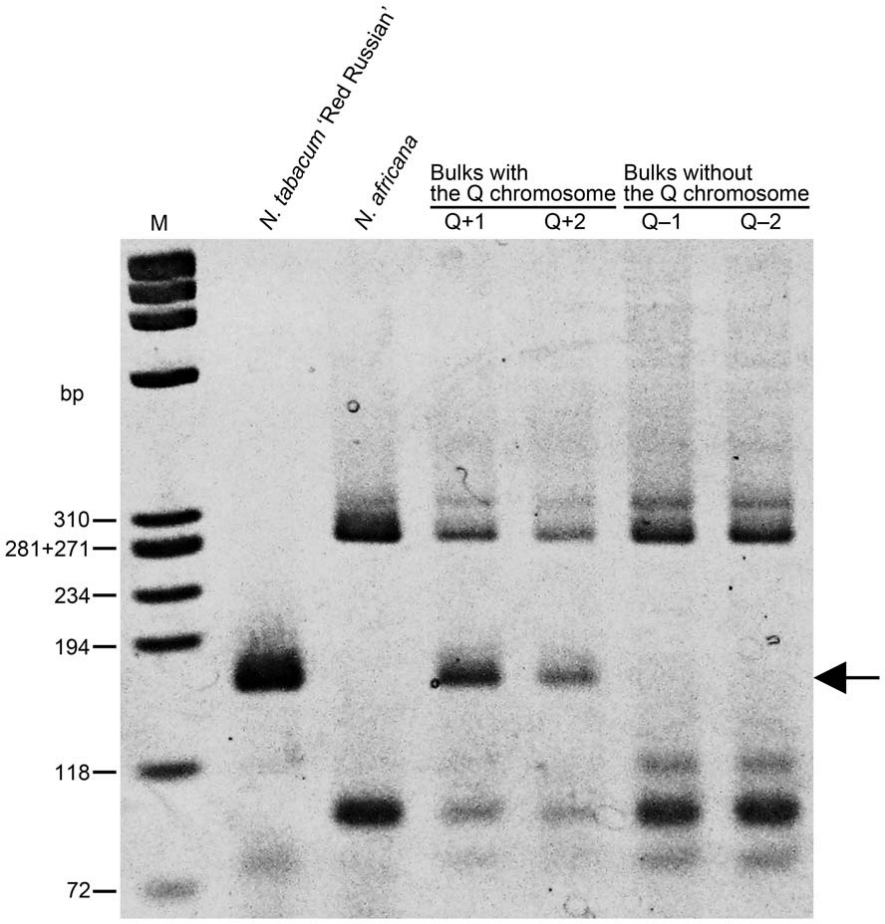
Detection of the SSR marker PT20383 in two types of DNA bulks. The marker band PT20383A representing the Q chromosome (Table 2) was amplified in *N. tabacum* ‘Red Russian’ and bulks Q+1 and Q+2, but not in other samples, as indicated by the arrow. M, size marker φX174/*Hae* III.

A linkage group corresponding to the Q chromosome was identified from a linkage map of *N. tabacum* [20]. First, we selected SSR markers at random from all linkage groups and checked whether they produced polymorphic bands between ‘Red Russian’ and *N. africana*. Most of the markers amplified one or two bands in each species, since both species are allotetraploid. Each band was named by the marker name followed by a letter. Many of these bands showed polymorphisms between ‘Red Russian’ and *N. africana* (Table 2). Next, we investigated whether the selected SSR markers produced polymorphic bands between the two types of DNA bulks. As a result, the SSR marker PT20383 on linkage group 11 was identified as showing polymorphism between the bulks (Table 2, Figure 1). Other SSR markers that mapped to linkage group 11 were investigated, and eight markers gave similar results to those of PT20383, indicating that linkage group 11 corresponds to the Q chromosome. This result was consistent with a report that the origin of markers in linkage group 11 is the S genome of *N. sylvestris*, an ancestor of *N. tabacum* [20], [24].

**Table 2:** Analysis of SSR markers in ‘Red Russian’, *N. africana* and two types of DNA bulks.

			Bands detected^c^			
SSR marker^a^	Linkage group^b^	Annealing temperature (°C)	‘Red Russian’	*N. africana*	Bulks with the Q chromosome	Bulks without the Q chromosome
PT30169	1	55	A, B	–	A, B	A, B
PT1085	2	55	A, B	B	A, B	A, B
PT20286	3	50	A	B, C	A, B, C	A, B, C
PT30138	3	55	A	B	A, B	A, B
PT30107	4	55	A	–	A	A
PT30361	4	55	A, B	–	A, B	A, B
PT30111	5	55	A, B	B	A, B	A, B
PT30229	6	55	A	B	A, B	A, B
PT1037	6	50	Multiple	Multiple	Not analyzed	Not analyzed
PT20172	6	55	A	B	A, B	A, B
PT30165	7	55	A	B	A, B	A, B
PT30485	8	55	A	B	A, B	A, B
PT30235	9	55	A	–	A	A
PT1078	10	55	A	–	A	A
PT30024	11	55	A	A, B	Not analyzed	Not analyzed
*PT30027*	11	50	A, B	B	A, B	B
*PT20383*	11	55	A	B, C	A, B, C	B, C
PT1245	11	55	A, B	B	A, B	A, B
*PT1348*	11	50	A	B	A, B	B
*PT1089*	11	50	A, B	–	A, B	B
*PT30420*	11	50	A	–	A	–
*PT30046*	11	55	A	B, C	A, B, C	B, C
PT30417	11	55	A	A	Not analyzed	Not analyzed
*PT30137*	11	50	A	B	A, B	B
PT30480	11	50	A, B	Multiple	Not analyzed	Not analyzed
PT1002	11	50	A, B	B	A, B	A, B
*PT30342*	11	55	A, B	B	A, B	B
*PT30365*	11	55	A, B	B, C	A, B, C	B, C
PT30063	12	55	A	–	A	A
PT30028	13	55	A	B	A, B	A, B
PT30249	13	55	A	A	Not analyzed	Not analyzed
PT30422	14	55	A	B	A, B	A, B
PT30016	15	55	A, B	–	A, B	A, B
PT20196	16	50	A	–	A	A
PT30159	17	55	A	–	A	A
PT30123	17	55	A	–	A	A
PT30243	18	50	A	B	A, B	A, B
PT54888	19	50	A, B	C	A, B, C	A, B, C
PT51696	19	50	A	B	A, B	A, B
PT30421	20	55	A	–	A	A
PT20388	21	55	A	–	A	A
PT1289	22	55	A, B	B	A, B	A, B
PT20445	23	55	A, B	B	A, B	A, B
PT30242	24	55	A, B	B	A, B	A, B
PT30375	24	55	A, B	B	A, B	A, B

a SSR markers identified as showing polymorphism between the two types of bulks are italicized.

b Bindler et al. [20].

c Different letters for each marker indicate bands with different sizes and the same letters indicate bands with the same size. A ‘–’ indicates that no band was detected.

Although the band termed PT1245A, detected by PT1245, showed polymorphism between ‘Red Russian’ and *N. africana*, the band showed no polymorphism between the two types of bulks (Table 2). Further studies using *N. ingulba* revealed that band PT1245A was not derived from the Q chromosome but another band, PT1245B, which showed no polymorphism between ‘Red Russian’ and *N. africana*, was derived from it (see below). Similarly, the polymorphic band PT1002A, detected by PT1002, showed no polymorphism between the two bulks (Table 2). Although another band, PT1002B, showed no polymorphism between ‘Red Russian’ and *N. africana*, or between ‘Red Russian’ and *N. ingulba*, this band was likely derived from the Q chromosome.

### Hybrid Lethality Observed in Crosses between *N. tabacum* and *N. ingulba*

**Figure 2:**
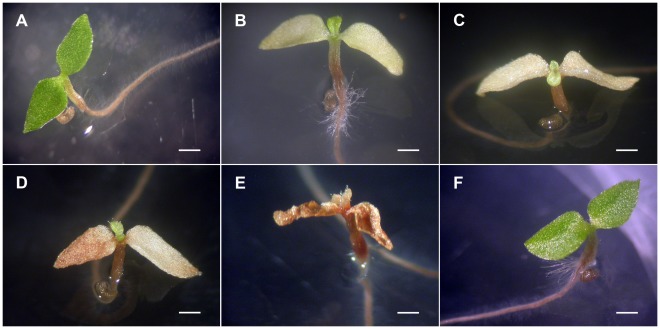
Lethal symptoms observed in hybrid seedlings from reciprocal crosses between *N. ingulba* and *N. tabacum*. When hybrid seedlings from the cross *N. ingulba* × *N. tabacum* ‘Red Russian’ were cultured at 28°C, the hypocotyl and roots turned brown 6 days after germination (DAG) (A). The cotyledons turned yellow at 20 DAG (B), and brown at 26 DAG (C). The young true leaves and shoot apices were still green at 43 DAG (D), but the seedlings turned completely brown and died at 51 DAG (E). Hybrid seedlings from the reciprocal cross ‘Red Russian’ × *N. ingulba* showed similar symptoms, browning of the hypocotyl and roots, at 13 DAG at 28°C (F). Scale bars = 1 mm.

Reciprocal crosses were carried out between ‘Red Russian’ and *N. ingulba* (section *Suaveolentes*) using conventional cross-pollination (Table 3). Although seeds were obtained from reciprocal crosses, the percentage of seed germination was significantly different: 82.5% when *N. ingulba* was used as the maternal parent and 5.7% when ‘Red Russian’ was used as the maternal parent. All hybrid seedlings from reciprocal crosses were inviable at 28°C (Figure 2). Characteristic symptoms of hybrid lethality were browning of the hypocotyl and roots, indicating that hybrid lethality in reciprocal crosses was Type II. The same results in reciprocal crosses suggested that hybrid lethality is due to the interaction of coexisting heterologous genomes, and not to a cytoplasmic effect.

**Table 3:** Viability of reciprocal hybrids between *N. tabacum* and *N. ingulba* at 28°C.

				Number of hybrids obtained			
Cross combination	Number of flowers pollinated	Number of capsules obtained	Number of seeds sown	Total	Viable	Inviable	Lethality type^a^
*N. ingulba* × ‘Red Russian’	20	18	120	99	0	99	II
‘Red Russian’ × *N. ingulba*	16	10	105	6	0	6	II

a Lethality types in *Nicotiana* were defined by Yamada et al. [1].

To investigate whether hybrid lethality is suppressed at elevated temperatures, seven hybrid seedlings from the cross *N. ingulba* × ‘Red Russian’ were obtained and cultured at 36°C. Hybrid lethality was completely suppressed at this temperature. However, when hybrid seedlings cultured at 36°C were transferred to 28°C, hybrid lethality was induced and all hybrid seedlings died (data not shown).

### The Q Chromosome Causes Hybrid Lethality in Crosses between *N. tabacum* and *N. ingulba*

To determine whether the Q chromosome causes hybrid lethality in the cross *N. tabacum* × *N. ingulba*, *N. ingulba* was crossed with *N. tabacum* monosomic plants derived from the cross Haplo-Q × ‘Samsun NN’ [18]. Those monosomic plants lacking the Q chromosome were used as maternal parents for the interspecific cross, since the transmission of the monosomic condition through pollen is very low [25].

Twenty-one flowers of monosomic plants were pollinated with *N. ingulba*. Four flowers produced capsules containing only powdery seeds and we were unable to obtain seeds with normal appearance. Since the percentage of flowers fertilized was low, we carried out test-tube pollination and ovule culture. Twenty placentas of monosomic plants were aseptically pollinated with *N. ingulba*. Sixteen hybrid seedlings were obtained from 189 ovules and cultured at 36°C. Four of these hybrid seedlings showed abnormal morphology and had no cotyledons and leaves. These four seedlings were excluded from further analysis.

**Figure 3:**
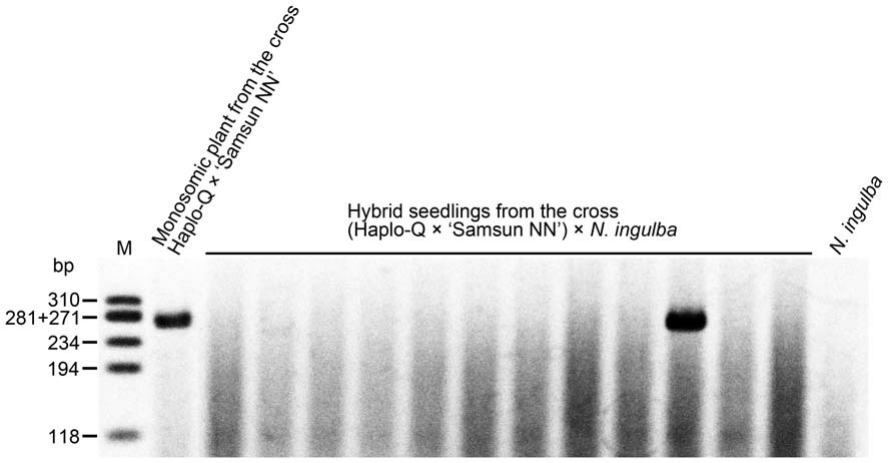
Detection of the SSR marker PT1348 in hybrid seedlings from the cross (Haplo-Q × ‘Samsun NN’) × *N. ingulba*. PT1348 was detected in one hybrid seedling but not in the other 11 hybrid seedlings. M, size marker φX174/*Hae* III.

Three Q-chromosome-specific STS markers, QCS2, QCS3 and QCS4, and 14 SSR markers mapped to linkage group 11 were analyzed. All STS and SSR markers excluding PT30024 produced polymorphic bands between parents, monosomic plants and *N. ingulba* (Table 4). When three of the STS and nine SSR markers that produced one polymorphic locus from monosomic plants were analyzed in hybrid seedlings, the bands detected by markers excluding PT1002 were observed in one hybrid seedling and not in the other 11 hybrid seedlings (Table 4, Figure 3). For three SSR markers, PT1245, PT30342 and PT30365, which produced two polymorphic bands from monosomic plants, one of the two bands was detected in one hybrid seedling and not in the other 11 hybrid seedlings, suggesting that the bands segregating in hybrid seedlings were specific to the Q chromosome (Table 4). Thus, using STS and SSR markers, 12 hybrid seedlings were divided into two types, one hybrid seedling possessing the Q chromosome and 11 hybrid seedlings lacking the Q chromosome.

**Table 4:** Analysis of SSR markers on linkage group 11 and Q-chromosome-specific STS markers in hybrids from the cross (Haplo-Q × ‘Samsun NN’) × *N. ingulba*.

		Bands detected^b^			
Marker	Map distance (cM)^a^	Monosomic plants from the crossHaplo-Q × ‘Samsun NN’^c^	*N. ingulba*	One inviable hybrid	Eleven viable hybrids
QCS2	Unmapped	A	–	A	–
QCS3	Unmapped	A	–	A	–
QCS4	Unmapped	A	–	A	–
PT30024	5.297	A	Multiple	Not analyzed	Not analyzed
PT30027	22.637	A, B	B	A, B	B
PT20383	38.629	A	–	A	–
PT1245	39.717	A, B	–	A, B	A
PT1348	49.011	A	–	A	–
PT1089	60.308	A, B	B	A, B	B
PT30420	68.146	A	–	A	–
PT30046	92.921	A	B	A, B	B
PT30417	109.58	A	–	A	–
PT30137	113.995	A	–	A	–
PT30480	115.629	A, B	B	A, B	B
PT1002	116.733	A, B	B	A, B	A, B
PT30342	121.984	A, B	–	A, B	B
PT30365	122.828	A, B	–	A, B	B

a Bindler et al. [20].

b Different letters for each marker indicate bands with different sizes and the same letters indicate bands with the same size. A ‘–’ indicates that no band was detected.

c The same bands for *N. tabacum* in Table 2 and Table 4 are indicated by the same letters.

Hybrid seedlings cultured at 36°C were transferred to 28°C. The one hybrid seedling possessing the Q chromosome died, while the 11 hybrid seedlings lacking the Q chromosome survived. This result indicated that one or more genes on the Q chromosome are responsible for hybrid lethality in the cross between *N. tabacum* and *N. ingulba*. Furthermore, we confirmed that linkage group 11 corresponds to the Q chromosome.

### Dissection of a Haplo-Q × *N. africana* Hybrid with a Possible Deletion of the Q Chromosome Segment

To identify the possibly deleted region of the Q chromosome in a viable hybrid seedling from the cross Haplo-Q × *N. africana* (in which QCS1, QCS3 and QCS4 were detected and QCS2 was not detected), seven SSR markers showing polymorphism between the two types of bulks derived from hybrids of *N. tabacum* × *N. africana* (Table 2) were tested. Two markers, PT30342 and PT30365, were not detected, while the other five markers were detected (Table 5). This indicated that the chromosomal region where PT30342 and PT30365 are located was deleted in the hybrid seedling.

**Table 5:** Analysis of a Haplo-Q × *N. africana* hybrid with a possible deletion of the Q chromosome segment using SSR markers on linkage group 11.

SSR marker	Map distance (cM)^a^	Marker detection^b^
PT30027	22.637	+
PT20383	38.629	+
PT1348	49.011	+
PT30046	92.921	+
PT30137	113.995	+
PT30342	121.984	–
PT30365	122.828	–

a Bindler et al. [20].

b A ‘+’ indicates that the marker band derived from the Q chromosome was detected and ‘–’ indicates that it was not.

## Discussion

Our previous studies revealed that many species of section *Suaveolentes* yield inviable hybrids after crosses with *N. tabacum* [14]–[16]. *N. ingulba*, which belongs to section *Suaveolentes*, is one species yielding inviable hybrids. In all these cases, hybrid lethality was Type II, which is suppressed at elevated temperatures ranging from 34 to 36°C, and the hybrid lethality gene(s) in *N. tabacum* were on the Q chromosome in the S subgenome. Recent studies based on internal transcribed spacer regions of rDNA, plastid genes, and ncpGS indicated that section *Suaveolentes* is a monophyletic group [11], [12], [26]. Based on this information, *Suaveolentes* species that yield inviable hybrids after crosses with *N. tabacum* should share the same gene triggering hybrid lethality by interaction with gene(s) on the Q chromosome. However, this conclusion is not applicable in all cases, since at least one species in section *Suaveolentes*, *N. occidentalis*, yields inviable hybrids even when they lack the Q chromosome [9].

The cross *N. tabacum* × *N. africana* rarely yields viable aneuploid hybrids or haploids [27], [28]. In a previous study, we obtained a viable aneuploid hybrid lacking the Q chromosome from the reciprocal cross [16]. In addition to these aneuploids and haploids, we revealed that viable hybrids possessing this chromosome with a deletion of certain regions can be obtained from crosses. Although such deletions might be observed in all chromosomes of reciprocal hybrids between *N. tabacum* and *N. africana*, deletion within the Q chromosome would be preferentially detectable, since gene(s) triggering hybrid lethality are located on the Q chromosome and most hybrids with an intact Q chromosome would die. A viable hybrid from the cross *N. tabacum* Haplo-Q × *N. africana* had a Q chromosome with a deletion of the region where PT30342 and PT30365 are located, suggesting that this region includes the causal gene(s) for hybrid lethality. This conclusion was drawn from only one hybrid plant with the deletion, since we could not obtain other deletion hybrids due to their rareness. Further studies will be needed to confirm and narrow down the position of the causal gene(s).

A linkage map is important for mapping, marker-assisted selection and map-based cloning of desirable genes. In *N. tabacum*, the first linkage map using SSR markers has been constructed [19], [20]. In the future, it will be desirable to assign linkage groups in the linkage map to each chromosome. For this purpose, monosomic lines should be useful, as demonstrated in the present study. Fortunately, a complete set of 24 monosomic lines of *N. tabacum* has been developed [21], [22]. Therefore, hybrid lines lacking certain chromosomes can be obtained through crosses between monosomic lines and *Nicotiana* species. *Nicotiana* species other than *N. tabacum* might be desirable for the crosses, since many polymorphisms would be observed, as in the present study. However, in such cases, production of hybrid lines may be problematic due to crossing barriers in interspecific crosses. Interestingly, the two *N. tabacum* cultivars used for the construction of the linkage map were ‘Red Russian’ and ‘Hicks Broad Leaf’ [19], [20]. Since the genetic background of all 24 monosomic lines of *N. tabacum* is ‘Red Russian’ [21], [22], ‘Hicks Broad Leaf’ would be the best parent to produce hybrid lines lacking a certain chromosome.

Another method to correlate linkage groups with chromosomes in *N. tabacum* has been reported by Vontimitta et al. [29], who mapped two genes responsible for the accumulation of *cis*-abienol and sucrose esters to a certain linkage group. Based on earlier monosomic analyses that revealed that both genes are located on the A chromosome [30], [31], Vontimitta et al. determined the correlation between the linkage group and the A chromosome. This indirect method is useful to assign linkage groups to each chromosome. However, the number of genes allocated to the chromosome by monosomic analyses is too small to determine all chromosomal assignments of linkage groups. On the other hand, our method using hybrid lines lacking a certain chromosome is theoretically applicable to all chromosomes in *N. tabacum*. This is the first report of a practical method to correlate linkage groups with chromosomes in *N. tabacum*.

## Materials and Methods

### Plant Materials

F_1_ seeds derived from crosses *N. tabacum* (2n = 48, SSTT) ‘Red Russian’ × *N. africana* (2n = 46) and *N. tabacum* Haplo-Q (2n = 47) × *N. africana* were obtained in a previous study [16] and used for bulked segregant analysis [23]. *N. ingulba* (2n = 40), which belongs to section *Suaveolentes*, was used for crosses with ‘Red Russian’ or F_1_ progeny (2n = 47) derived from the cross Haplo-Q × *N. tabacum* ‘Samsun NN’; the latter was identified by the Q-chromosome-specific STS marker QCS1 [18]. All plants used as parents were cultivated in a greenhouse.

### Interspecific Crosses

Flowers of plants used as female parents were emasculated 1 day before anthesis and pollinated with pollen of plants used as male parents. F_1_ seeds were sterilized with 5% sodium hypochlorite for 15 min. The sterilized seeds were sown in Petri dishes (60 mm diameter, 17 mm deep) containing 8 ml of 1/2 MS medium [32] supplemented with 1% sucrose and 0.2% Gelrite (pH 5.8) and then cultured at 28°C under continuous illumination (approximately 150 µmol m^−2^ s^−1^).

Test-tube pollination in combination with ovule culture was carried out as previously described [33]. Anthers of plants used as male parents were aseptically excised from still-closed flowers and stimulated to dehisce in an incubator (28°C). Flowers of plants used as female parents were emasculated 1 day before anthesis. On the next day, flowers of plants used as female parents were collected and their corolla, sepals and styles were removed. The ovaries were surface-sterilized with 70% ethanol for 30 s and with 5% sodium hypochlorite solution for 5 min. The ovary walls were peeled back to expose the placentas with intact ovules, and then the ovaries were placed in Petri dishes containing 8 ml of medium with 3% sucrose and 0.8% agar (pH 5.8). Pollen of plants used as male parents was spread on the surface of the placentas. Pollinated placentas were maintained at 28°C under continuous illumination. Fertilized and enlarged ovules were excised from placentas at 10 to 14 days after pollination and cultured in Petri dishes containing 8 ml of 1/2 MS medium supplemented with 3% sucrose and 0.8% agar (pH 5.8) at 28°C under continuous illumination.

### Cultivation of Hybrid Seedlings

Hybrid seedlings were cultured at 28°C under continuous illumination. Some seedlings were transferred to flat-bottomed test tubes (25 mm diameter, 100 mm length) that contained 10 ml of 1/2 MS medium supplemented with 1% sucrose and 0.2% Gelrite (pH 5.8) immediately after germination and were cultured at 36°C under continuous illumination. Hybrid seedlings cultured at 36°C for 30 days after germination were transferred to 28°C under continuous illumination.

### PCR Analysis

Total DNA was extracted from leaves of each plant using a cetyltrimethylammonium bromide method [34]. Q-chromosome-specific STS markers, QCS1, QCS2, QCS3 and QCS4, were detected as previously described [14], [18].

Forty-five SSR markers (Table 2) covering all linkage groups were detected as follows. Reaction mixtures contained 1× ThermoPol reaction buffer (New England Biolabs, Tokyo, Japan), 0.2 mM of each dNTP, 0.2 µM of each primer, 20 ng of template DNA, 0.5 U of *Taq* DNA polymerase (New England Biolabs) in a total volume of 20 µl. PCR amplification was performed using the TProfessional Basic Thermocycler (Biometra, Göttingen, Germany) programmed for 3 min at 94°C for initial denaturation, followed by 35 cycles of 30 s at 94°C, 30 s at 50 or 55°C, 1 min at 72°C, and a final extension of 5 min at 72°C. PCR products were separated by electrophoresis in a 3% agarose gel in TBE buffer and stained with ethidium bromide.
